# Genome-wide association study and genomic selection for soybean chlorophyll content associated with soybean cyst nematode tolerance

**DOI:** 10.1186/s12864-019-6275-z

**Published:** 2019-11-27

**Authors:** Waltram Second Ravelombola, Jun Qin, Ainong Shi, Liana Nice, Yong Bao, Aaron Lorenz, James H. Orf, Nevin D. Young, Senyu Chen

**Affiliations:** 1 Department of Horticulture, PTSC316, University of Arkansas, Fayetteville, AR 72701 USA; 2 Hebei Cereal & Oil Crop Institute, Hebei Academy of Agricultural and Forestry Sciences, Shijiazhuang, 050031 Hebei China; 3 Southern Research & Outreach Center, University of Minnesota, Waseca, MN 56093 USA; 4 Department of Agronomy and Plant Genetics, University of Minnesota, St. Paul, MN 55108 USA; 5 Department of Plant Pathology, University of Minnesota, St. Paul, MN 55108 USA

**Keywords:** Genome-wide association study (GWAS), Soybean cyst nematode (SCN), Leaf chlorophyll content, Single nucleotide polymorphism (SNP), Genomic selection (GS)

## Abstract

**Background:**

Soybean cyst nematode (SCN), *Heterodera glycines* Ichinohe, has been one of the most devastating pathogens affecting soybean production. In the United States alone, SCN damage accounted for more than $1 billion loss annually. With a narrow genetic background of the currently available SCN-resistant commercial cultivars, high risk of resistance breakdown can occur. The objectives of this study were to conduct a genome-wide association study (GWAS) to identify QTL, SNP markers, and candidate genes associated with soybean leaf chlorophyll content tolerance to SCN infection, and to carry out a genomic selection (GS) study for the chlorophyll content tolerance.

**Results:**

A total of 172 soybean genotypes were evaluated for the effect of SCN HG Type 1.2.3.5.6.7 (race 4) on soybean leaf chlorophyll. The soybean lines were genotyped using a total of 4089 filtered and high-quality SNPs. Results showed that (1) a large variation in SCN tolerance based on leaf chlorophyll content indices (CCI); (2) a total of 22, 14, and 16 SNPs associated with CCI of non-SCN-infected plants, SCN-infected plants, and reduction of CCI SCN, respectively; (3) a new locus of chlorophyll content tolerance to SCN mapped on chromosome 3; (4) candidate genes encoding for Leucine-rich repeat protein, plant hormone signaling molecules, and biomolecule transporters; and (5) an average GS accuracy ranging from 0.31 to 0.46 with all SNPs and varying from 0.55 to 0.76 when GWAS-derived SNP markers were used across five models. This study demonstrated the potential of using genome-wide selection to breed chlorophyll-content-tolerant soybean for managing SCN.

**Conclusions:**

In this study, soybean accessions with higher CCI under SCN infestation, and molecular markers associated with chlorophyll content related to SCN were identified. In addition, a total of 15 candidate genes associated with chlorophyll content tolerance to SCN in soybean were also identified. These candidate genes will lead to a better understanding of the molecular mechanisms that control chlorophyll content tolerance to SCN in soybean. Genomic selection analysis of chlorophyll content tolerance to SCN showed that using significant SNPs obtained from GWAS could provide better GS accuracy.

## Key message

To the best of our knowledge, this is the first report of QTL associated with chlorophyll content tolerance to soybean cyst nematode (SCN) in soybean.

## Background

Soybean [*Glycine max* (L.) Merr.] is one of the most important legumes worldwide by providing oil and being a source of vegetable protein. Developing soybean-derived biofuel has been recently increasing, with an estimated value exceeding $35 billion in the United States (www.soystats.com). Soybean cyst nematode (SCN), *Heterodera glycines* Ichinohe, is an important pest with total annual yield losses about $1.5 billion in the U.S. alone [1]. The SCN is an obligate endoparasite, which feeds on soybean roots, depletes carbon of soybean plants and results in yield losses [2]. One pathway of SCN damage to soybean is induction or enhancement of nutritional deficiency of soybean such as iron, potassium, and/or nitrogen deficiencies that result in chlorophyll content reduction or in severe cases the typical chlorosis symptom [3, 4]. Iron-deficiency chlorosis (IDC) of soybean, in particular, is common in the North Central region, the major soybean production region in the USA. It occurs in high pH soil, but many biotic and abiotic factors affect its occurrence [5–8]. The SCN is present in most soybean fields in the region, and high pH also favors reproduction of SCN and its damage to soybean plants [9]. Therefore managing SCN and nutritional deficiencies is important for soybean productivity in many fields in the North Central USA and some other regions in the world.

Use of SCN-resistant soybean cultivars and crop rotation involving a non-host crop is the best way to manage SCN [10, 11]. Development of new SCN-resistant soybean cultivars requires a better understanding of the genetic mechanisms underlying SCN resistance. To date, at least 216 SCN-resistant QTL have been reported (www.soybase.org). A large number of those QTL have not been fully investigated [12]. Among the QTL conferring resistance to SCN, two loci, *rhg1* and *Rhg4*, which are located on chromosomes 18 and 8, respectively, have been commonly used to deploy SCN resistance in soybean germplasm [13]. Both *rhg1* and *Rhg4* are required in the soybean cultivar ‘Forest’ to exhibit resistance to SCN, with *Rhg4* being dominant [14]. This resistance has been known as Peking-type resistance because the source of resistance was from Peking. In contrast, the resistance in cultivars with PI 88788 source requires only *rhg1*, and the resistance is known as PI 88788-type [15].

Some studies of the genetic mechanism between the two aforementioned SCN-resistant loci have been reported. A gene mapped at the *Rhg4* locus and conferring SCN resistance has been cloned [16]. This gene encodes for a serine hydroxymethyltransferase [16]. The SCN-resistant gene within the *Rhg4* locus was derived from an artificial selection occurring during soybean domestication [17]. Resistance to SCN conferred by the *rhg1* locus has been associated to copy number variation and DNA methylation, which can enhance the expression of SCN resistance genes within that locus [18]. Three genes in the *rhg 1* locus encoding an amino acid transporter, an α-SNAP protein, a WI12 (wound-inducible domain) protein contribute to the SCN resistance [19, 20].

The utilization of molecular markers through marker-assisted selection (MAS) in soybean breeding programs has been proven to accelerate the development of disease-resistant cultivars [21]. Recently, tools such as genome-wide association mapping (GWAS) and genomic selection (GS) have increasingly become popular in efforts towards uncovering the genetic basis of traits of interest in agriculture and identifying important new loci. GWAS has been used to identify new markers and loci associated with resistance to SCN. A total of 6 SSR markers associated with SCN resistance were identified in a set of 159 soybean lines [22]. GWAS was conducted on a total of 282 soybean genotypes to identify SNP markers associated with resistance to SCN HG type 0 [12]. Out of the 1536 SNPs used, a total of 7 SNP markers were associated with SCN resistance. Most of those significant SNP markers were located in the *rhg1* locus. In addition, two genes, *FGAM1* and *Glyma18g46201*, were located in the vicinity of two significant SNPs. A total of 19 SNP markers were reported to be associated with resistance to SCN HG type 0 and HG type 1.2.3.5.7 in an association panel consisting of 440 soybean genotypes, of which, three were mapped to loci that have not yet been reported [23]. A total of 553 soybean genotypes were evaluated for resistance to SCN HG type 0 and GWAS allowed for the discovery of 8 new loci associated with SCN on this association panel [24].

Genomic selection has been frequently used to achieve faster genetic gain in plant breeding [25]. Genomic selection has often been proven to have superior features over the traditional MAS when dealing with complex traits [12]. In the earliest genomic selection study on resistance to SCN [12], genomic selection accuracy for the SCN resistance was in the range of 0.59 to 0.67.

The objectives of this study were (i) to conduct a genome-wide association study to identify QTL associated with leaf chlorophyll content in soybean in SCN infested and non-infested soils, and the QTL associated with reduction of chlorophyll content by SCN; (ii) identify SNP markers and candidate genes associated with the traits; (iii) to carry out a genomic selection study for tolerance of soybean chlorophyll content to SCN infection.

## Results

### Chlorophyll content phenotyping associated with SCN

**Fig. 1:**
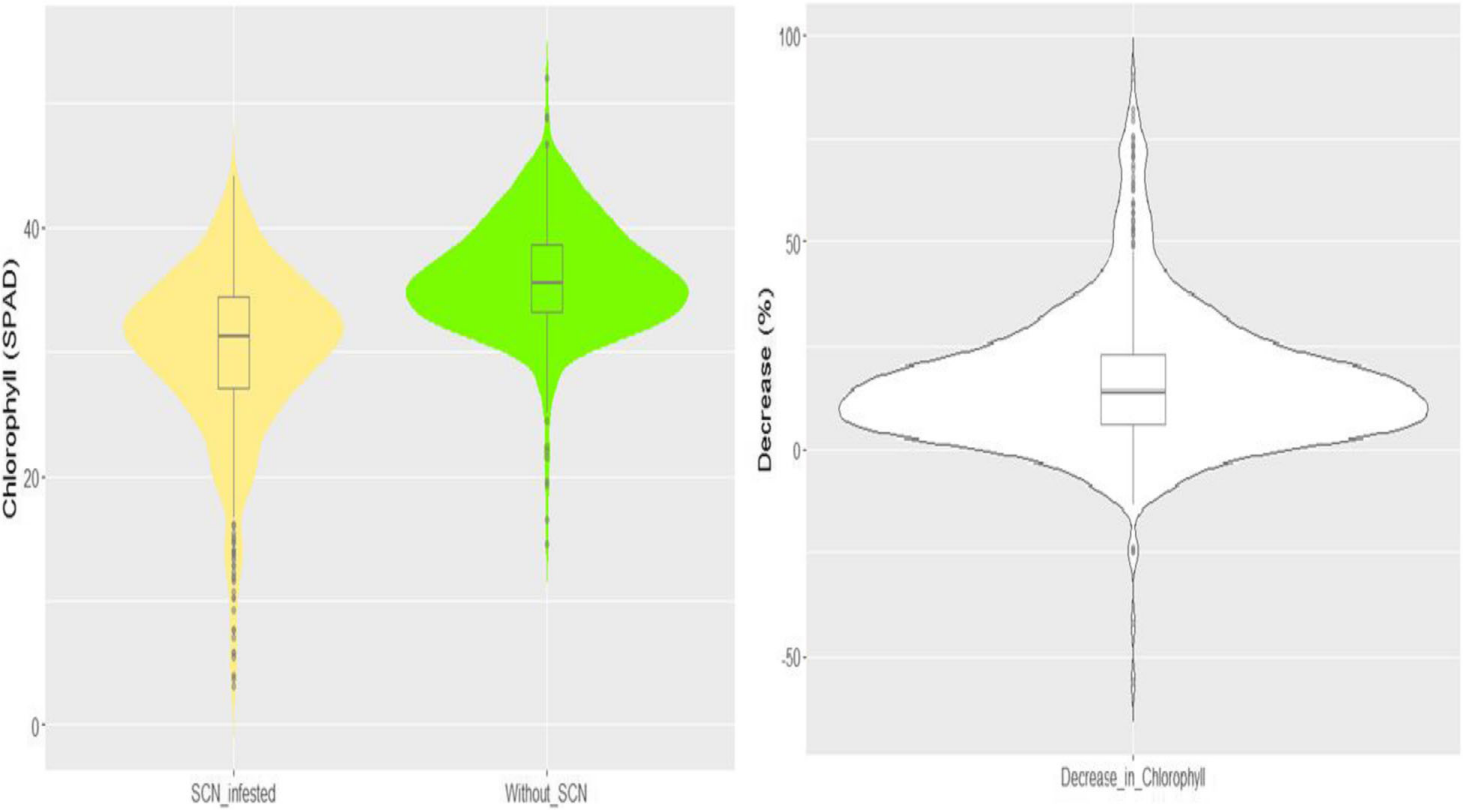
Combined violin-boxplots representing the probability density function of leaf chlorophyll content indices for plants grown in SCN-infested soils (yellow), plants grown in soils without SCN (green), and percentage reduction in leaf chlorophyll content indices due to SCN

Soybean leaf chlorophyll content (CCI) in non-SCN-infestation recorded at 8 weeks after planting was significantly different among the genotypes (F-value = 11.17, *p*-value< 0.0001) (Table 1). The CCI was approximately normally distributed (Fig. 1). The genotypes having the highest CCI on non-SCN-infested soils were MN0082SP (48.3), GRANDE (44.1), MN0603SP (43.9), AGASSIZ (43.5), M98240104 (43.4), MN1011CN (43.3), MN0502 (43.0), MN1106CN (43.0), CHICO (42.7), and WALSH (42.6) (Additional file 1: Table S1). Those having the lowest CCI were HARK (31.3), MN1008SP (31.2), VINTON81 (30.8), M97205096 (30.5), KATO (30.2), PI372403A (29.8), M95118009 (29.3), PI437228 (24.4), PI257428 (22.7), and NORMAN (22.6) (Additional file 1: Table S1).

**Table 1:** ANOVA for leaf chlorophyll content of plants without SCN, plants infested with SCN, and decrease in chlorophyll content due to SCN

Traits	Source	DF	Sum of Squares	Mean Square	F Value	Pr > F
Without SCN	Genotype	171	10,460.76	63.02	11.17	<.0001
	Error	516	2939.98	5.64		
SCN-infested	Genotype	171	23,423.78	141.11	9.43	<.0001
	Error	516	7791.98	14.96		
Decrease in chlorophyll (%)	Genotype	171	110,482.93	665.56	4.26	<.0001
	Error	516	81,465.40	156.36

The distribution of CCI of soybean in the SCN-infested soil was nearly normal (Fig. 1). Significant differences in CCI in the SCN-infected plants were found among the genotypes (F-value = 9.43, *p*-value< 0.0001) (Table 1). The genotypes exhibiting high CCI under SCN infestation were MN1011CN (41.5), M98134022 (41.2), MN1106CN (40.4), M98240104 (40.3), AGASSIZ (40.0), GRANDE (39.1), LAMBERT (38.2), SWIFT (38.1), CHICO (38.0), and MN0502 (37.5) (Additional file 1: Table S1). The lowest CCI under SCN infestation was found for the genotypes PI257428 (19.2), MN1607SP (18.9), PI437267 (17.3), MN1307SP (15.7), MN1406SP (15.2), MN1008SP (15.2), PORTAGE (14.9), MN1603SP (14.0), NORMAN (9.1), and PI437228 (8.1) (Additional file 1: Table S1). Of the top 10 genotypes having the highest CCI under non-SCN infestation, 7 (MN1011CN, MN1106CN, M98240104, AGASSIZ, GRANDE, CHICO, and MN0502) had the highest CCI when grown in SCN-infested soils. Of the 10 genotypes grown in SCN free soils and having the lowest CCI, 4 (PI257428, MN1008SP, NORMAN, and PI437228) still showed the lowest CCI when grown in SCN-infested soils.

Tolerance to SCN based on CCI was assessed by computing the percentage reduction in CCI due to SCN infection. Percentage reduction in CCI by SCN was approximately normally distributed (Fig. 1). On average, CCI was 36.0 in non-infested soil, and 30.1 in the SCN-infested soil, a 6.3% reduction. ANOVA showed significant differences in CCI reduction by SCN among the soybean genotypes (F-value = 4.26, *p*-value< 0.0001) (Table 1). CCI was almost not affected by SCN for the genotypes M99209070 (0.51%), M99286050 (0.58%), DWIGHT (0.88%), CHIPPEWA64 (1.14%), MN0203SP (1.86%), MN0201 (1.89%), MN0205SP (2.26%), M98134022 (2.32%), BURLISON (2.56%), and M99337034 (2.57%) (Additional file 1: Table S1), indicating that the leaf chlorophyll content of these genotypes was not sensitive to SCN infection. CCI of the genotypes PI437228 (66.87%), NORMAN (60.00%), MN1603SP (57.47%), PORTAGE (57.04%), MN1307SP (54.59%), MN1406SP (54.19%), PI437267 (52.66%), MN1008SP (51.40%), PI437994 (44.97%), and MN1007SP (44.26%) (Additional file 1: Table S1) were the most affected by SCN, suggesting that the leaf chlorophyll content of these genotypes could be highly sensitive to SCN infection. Pearson’s correlation coefficient between reduction in CCI and CCI without SCN was − 0.24. However, the correlation between reduction in CCI and CCI with SCN was − 0.85.

### SNP profile

A total of 4089 high-quality SNPs were used for genome-wide association analysis. The average SNP number per chromosome was in the range of 144 to 269 SNPs, with an average of 204. Chromosome 11 with 144 SNPs had the lowest number of SNPs, whereas chromosome 18 with 269 SNPs had the highest number of SNPs (Table 2). The average distance between two SNPs per chromosome varied from 119 kb to 352 kb, with an average of 251 kb. The shortest average distance between SNPs was found on chromosome 15, whereas the longest one was on chromosome 11 (Table 2). Average minor allele frequency (MAF) per chromosome ranged between 16.14 and 24.80%, with an average of 21.57% (Table 2). Percentage of heterozygous SNPs per chromosome was in the range of 7.57 to 10.76%, and averaging 9.30% (Table 2). Percentage of missing SNP per chromosome varied from 4.16 to 5.60%, with an average of 4.96% (Table 2).

**Table 2:** Distribution of SNPs obtained from the Soy6K SNP Infinium Chips, average distance between SNPs within each chromosome, average minor allele frequency, average percentage of heterozygous SNP, and average percentage of missing data per SNP

Chromosome	SNP_ Number	Average_distance_betweenSNP (kb)	MAF(%)^a^	H(%)^b^	Missing(%)^c^
1	159	352	19.17	9.21	4.79
2	254	223	23.51	9.72	5.30
3	194	267	22.65	9.40	5.15
4	190	286	24.25	9.43	5.09
5	194	239	23.38	10.02	5.10
6	205	275	19.96	9.23	4.38
7	215	189	16.72	8.39	4.67
8	225	208	21.98	8.21	4.57
9	191	274	24.80	8.93	5.03
10	216	280	23.78	9.57	5.37
11	144	266	16.14	7.57	4.16
12	174	256	21.48	8.52	4.79
13	262	201	21.01	9.64	4.86
14	196	302	21.91	9.80	4.59
15	235	119	24.24	10.76	5.60
16	165	227	22.76	9.43	5.35
17	197	235	22.44	10.23	5.13
18	269	291	18.81	8.91	4.93
19	200	279	21.23	8.58	5.02
20	204	260	21.24	10.50	5.27

^a^Minor Allele Frequency (MAF)

^b^Average percentage of heterozygous SNP

^c^Average percentage of missing SNP data

### Genome-wide association study (GWAS)

**Fig. 2:**
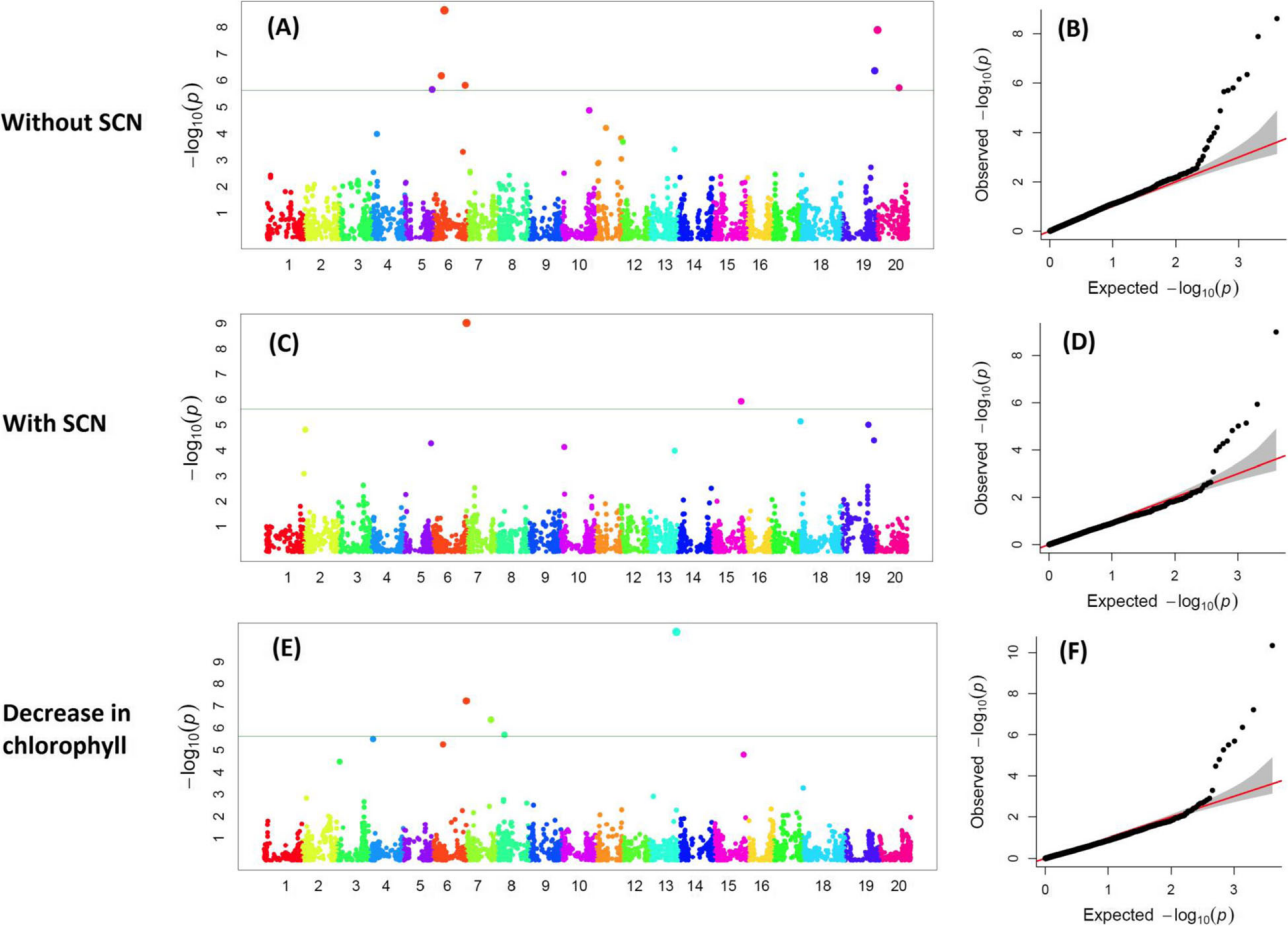
Graphs showing Manhattan plots and QQ-plots for leaf chlorophyll content indices (CCI) of plants non-infected by SCN, CCI plants infected by SCN, and reduction in CCI by SCN. **a** Manhattan plot for CCI of plants without SCN, **b** QQ-plot for CCI of the non-infected plants, **c** Manhattan plot for CCI of the SCN-infected plants, **d** QQ-plot for CCI of the SCN-infected plants, **e** Manhattan plot for reduction in CCI by SCN, and (**f**): QQ-plot for reduction in CCI

Genome-wide association study was conducted to identify SNPs associated with CCI under non-SCN infection, CCI in SCN-infected plants, and reduction in CCI by SCN. The number of significant SNPs varied among those aforementioned traits. A total of 22 SNPs were found to be significantly associated with CCI under non-infested condition. These SNPs were located on chromosomes 4, 5, 6, 7, 10, 11, 12, 13, 19, and 20 (Table 3). Of the 22 SNPs, five were found on chromosome 11 and 4 mapped on chromosome 6 (Fig. 2a). The QQ-plot showed that the model used to assess the SNPs was robust (Fig. 2b). Among the 22 SNPs associated with CCI for the non-infested plants, LOD varied from 2.51 to 8.63, with an average of 4.32 (Table 3). The SNPs having the highest LOD values were Gm06_16,792,113_T_C (8.63), Gm20_1,621,036_T_C (7.90), Gm19_48,074,289_A_C (6.35), Gm06_11,948,808_G_A (6.16), Gm06_47,439,414_C_T (5.80), Gm20_33,580,029_C_T (5.70), Gm05_40,299,923_A_G (5.65) (Table 3). Most of these high LOD value SNPs (LOD > 6) were located on chromosome 6 indicative of significant QTL associated with plant chlorophyll on this chromosome.

**Table 3:** Significant SNPs associated with leaf chlorophyll content for plants without SCN infestation, leaf chlorophyll content for SCN-infested plants, decrease in leaf chlorophyll content due to SCN, genes within 10 kb genomic region harboring the SNPs, and functional annotation of the genes

Trait	SNP_ID	Chromosome	Position (bp)	MAF (%)	LOD (−log10(*p*-value))^a^	Gene name^b^	Functional annotation
Leaf chlorophyll content undernon-SCN infestation	Gm04_2,574,201_T_G	4	2,574,201	14.11	2.54	Glyma.04 g032100	Predicted membrane protein
	Gm04_7,672,403_A_G	4	7,672,403	39.88	3.99	Glyma.04 g088800	Serine/threonine protein kinase
	Gm05_40,299,923_A_G	5	40,299,923	7.1	5.65	Glyma.05 g224000	Aspartyl/lysyl-trna synthetase
	Gm06_11,948,808_G_A	6	11,948,808	31.25	6.16	Glyma.06 g146400	4-alpha-glucanotransferase
	Gm06_16,792,113_T_C	6	16,792,113	6.62	8.63	Glyma.06 g191200	IQ-domain 31
	Gm06_43,980,786_G_A	6	43,980,786	6.02	3.31	NA^c^	NA
	Gm06_47,439,414_C_T	6	47,439,414	35.58	5.80	Glyma.06 g285800	Vascular plant one zinc finger protein
	Gm07_3,953,270_T_C	7	3,953,270	38.51	2.57	Glyma.07 g047100	Calcineurin-like metallo-phosphoesterase superfamily protein
	Gm07_3,990,308_A_G	7	3,990,308	37.42	2.52	Glyma.07 g047600	Chlorophyll A-B binding protein
	Gm10_4,458,104_G_A	10	4,458,104	30.62	2.51	Glyma.10 g049600	ROP interactive partner 3
	Gm10_41,610,215_C_T	10	41,610,215	17.58	4.88	Glyma.10 g183000	Phytoene dehydrogenase
	Gm11_3,641,716_A_C	11	3,641,716	26.41	2.87	Glyma.11 g048600	Formin-related
	Gm11_4,702,578_C_A	11	4,702,578	25.95	2.89	Glyma.11 g062300	Homeobox protein transcription factors
	Gm11_15,558,504_T_C	11	15,558,504	21.81	4.21	Glyma.11 g164300	Serine/threonine protein phosphatase
	Gm11_37,978,746_G_T	11	37,978,746	11.11	3.82	NA	NA
	Gm11_38,183,607_G_A	11	38,183,607	13.09	3.04	LOC106795218	NA
	Gm12_1,460,019_T_C	12	1,460,019	12.65	3.68	Glyma.12 g020500	2-C-methyl-D-erythritol 4-phosphate cytidylyltransferase
	Gm13_38,032,737_G_A	13	38,032,737	38.6	3.40	Glyma.13 g279200	Asparagine synthetase
	Gm19_42,195,616_G_A	19	42,195,616	28.05	2.72	Glyma.19 g161200	Uridine kinase
	Gm19_48,074,289_A_C	19	48,074,289	40	6.35	Glyma.19 g229800	Karyopherin (importin) alpha
	Gm20_1,621,036_T_C	20	1,621,036	26.06	7.90	Glyma.20 g017100	Sulfate transporter
	Gm20_33,580,029_C_T	20	33,580,029	16.97	5.70	Glyma.20 g092200	40S ribosomal protein
Leaf chlorophyll conent for SCN-infested plants	Gm02_207,506_A_G	2	207,506	4.76	3.09	Glyma.02 g001700	Protein of unknown function
	Gm02_2,246,479_A_G	2	2,246,479	33.33	4.82	Glyma.02 g025200	Protein of unknown function
	Gm03_36,634,361_G_A	3	36,634,361	5.36	2.64	Glyma.03 g151400	NA
	Gm05_39,995,603_C_T	5	39,995,603	7.74	4.27	Glyma.05 g220300	Formin binding protein and related proteins
	Gm06_50,593,128_T_G	6	50,593,128	22.84	9.01	Glyma.06 g317100	Predicted transporter
	Gm07_11,956,773_T_C	7	11,956,773	34.18	2.54	Glyma.07 g114300	Ethylene-responsive element binding factor 13
	Gm10_6,196,864_T_G	10	6,196,864	34.18	4.13	Glyma.10 g064900	Sequence-specific DNA binding transcription factors
	Gm13_39,378,998_G_A	13	39,378,998	5.81	3.97	Glyma.13 g294200	Putative signaling peptide similar to TAX1
	Gm14_49,357,738_A_G	14	49,357,738	6.55	2.52	NA	NA
	Gm15_43,797,502_G_T	15	43,797,502	23.75	5.94	Glyma.15 g233100	Leucine-rich repeat-containing protein
	Gm18_1,620,585_T_C	18	1,620,585	9.2	5.15	Glyma.18 g022100	BTB/POZ domain-containing protein
	Gm19_38,917,571_A_G	19	38,917,571	19.02	2.60	Glyma.19 g129700	F-box family protein
	Gm19_39,863,286_G_T	19	39,863,286	24.69	5.02	Glyma.19 g137300	Det1 complexing ubiquitin ligase
	Gm19_48,074,289_A_C	19	48,074,289	40	4.39	Glyma.19 g229800	Karyopherin (importin) alpha
Decrease in chlorophyll content	Gm02_6,340,233_C_A	2	6,340,233	4.19	2.83	Glyma.02 g072300	Methyltransferase-like protein
	Gm03_3,334,303_C_A	3	3,334,303	35.03	4.47	Glyma.03 g029900	Cytochrome P450
	Gm03_39,574,966_T_C	3	39,574,966	27.85	2.67	Glyma.03 g183700	NA
	Gm04_5,172,181_A_G	4	5,172,181	23.27	5.50	Glyma.04 g062600	NA
	Gm06_16,315,206_A_G	6	16,315,206	39.26	5.26	Glyma.06 g187300	Lipase (class 3)
	Gm06_50,593,128_T_G	6	50,593,128	22.84	7.22	Glyma.06 g317100	Predicted transporter
	Gm07_35,908,169_T_C	7	35,908,169	17.5	6.37	Glyma.07 g191600	Secretory carrier membrane protein
	Gm08_9,848,168_T_C	8	9,848,168	4.71	2.69	Glyma.08 g127700	Phosphatidylinositol-4-phosphate 5-kinase
	Gm08_10,116,360_C_T	8	10,116,360	5.32	2.75	Glyma.08 g132000	Protein of unknown function
	Gm08_11,501,419_A_C	8	11,501,419	5.36	5.70	Glyma.08 g149800	Iron/ascorbate family oxidoreductases
	Gm08_43,787,988_G_A	8	43,787,988	12.05	2.60	Glyma.08 g318600	NA
	Gm09_6,664,095_T_C	9	6,664,095	38.22	2.50	LOC106794327	NA
	Gm13_5,211,326_T_C	13	5,211,326	12.12	2.90	NA	NA
	Gm13_39,378,998_G_A	13	39,378,998	5.81	10.33	Glyma.13 g294200	Putative signaling peptide similar to TAX1
	Gm15_43,797,502_G_T	15	43,797,502	23.75	4.79	Glyma.15 g233100	Leucine-rich repeat-containing protein
	Gm18_1,427,298_G_T	18	1,427,298	5.29	3.29	Glyma.18 g019300	Copper transport protein ATOX1-related

^a^*p*-value associated to each SNP was obtained using the FarmCPU model

^b^Gene name was retrieved from Soybase using the *Glycine max* genome version Glyma.Wm82.a2 (Gmax2.0)

^c^Information was not available

Results showed a total of 14 SNPs significantly associated with leaf chlorophyll content for SCN-infested plants. These SNPs were found on chromosomes 2, 3, 5, 6, 7, 10, 13, 14, 15, 18, and 19. Of the 14 SNPs, 3 were mapped on chromosome 19 and 2 were identified on chromosome 2 (Fig. 2c). The QQ-plot suggested that the model used for identifying SNPs was reasonable (Fig. 2d). LOD values pertaining to those 14 SNPs were in the range of 2.52 to 9.01, with an average of 4.29 (Table 3). SNPs having the highest LOD values were Gm06_50,593,128_T_G (9.01), Gm15_43,797,502_G_T (5.94), Gm18_1,620,585_T_C (5.15), Gm19_39,863,286_G_T (5.02), Gm02_2,246,479_A_G (4.82) (Table 3), which were located on chromosomes 6, 15, 18, 19, and 2 (Fig. 2c).

A total of 16 SNPs were found to be associated with reduction in CCI due to SCN. Those SNPs were located on chromosomes 2, 3, 4, 6, 7, 8, 9, 13, 15, and 18 (Fig. 2e). Of the 16 SNPs, 4 were found on chromosome 8, suggesting significant QTL associated with tolerance to SCN in this region, based upon the reduction in CCI. The QQ-plot (Fig. 2f) indicated the robustness of the model used for GWAS. For the 16 SNPs, LOD values varied from 2.50 to 10.33, with an average of 4.49 (Table 3). The SNPs with the highest LOD values were Gm13_39,378,998_G_A (10.33), Gm06_50,593,128_T_G (7.22), Gm07_35,908,169_T_C (6.37), Gm08_11,501,419_A_C (5.70), Gm04_5,172,181_A_G (5.50), and Gm06_16,315,206_A_G (5.26) (Table 3), which were found on chromosomes 13, 6, 7, 8, 4, and 6, respectively. Two of the most significant SNPs were located on chromosome 6, indicating probable QTL affecting SCN on this region.

An overlapping significant SNP, Gm19_48,074,289_A_C, was found to be associated with both leaf chlorophyll content for non-SCN-infested and SCN-infested plants (Table 3). Three overlapping significant SNPs, Gm06_50,593,128_T_G, Gm13_39,378,998_G_A, and Gm15_43,797,502_G_T, were also identified for leaf chlorophyll content of plants grown in soils with SCN and the reduction in CCI (Table 3), indicating these SNP markers may not be related to SCN tolerance. However, no overlapping SNPs were identified for the traits leaf chlorophyll content under non-SCN infestation and reduction in CCI due to SCN, suggesting that these SNP markers were associated with SCN tolerance.

### Candidate genes

Genes within the 10 kb-genomic region flanking a significant SNP were taken into a consideration. Of the 22 SNPs significantly associated with leaf chlorophyll content under non-SCN infestation, 20 harbored genes within the 10 kb-flanking region (Table 3). Functional annotations pertaining to these candidate genes consisted of membrane proteins, kinase, phosphatase, biomolecule transferase, transporters, and transcription factors. The genomic region containing the significant SNP, Gm07_3,990,308_A_G, contained the gene *Glyma.07 g047600*, which encoded for a chlorophyll A-B binding protein and was directly involved in the chlorophyll pathway, which was indicative of the robustness and reliability of the SNPs reported in this current investigation (Table 3). In addition, the protein, 4-alpha-glucanotransferase, encoded by *Glyma.06 g146400* and widely found in photosynthetic leaves was also identified. Genes located within the 10-kb genomic region of the most significant SNPs, Gm06_16,792,113_T_C, Gm20_1,621,036_T_C, Gm19_48,074,289_A_C, Gm06_11,948,808_G_A, Gm06_47,439,414_C_T, and Gm20_33,580,029_C_T, were *Glyma.06 g191200*, *Glyma.20 g017100*, *Glyma.19 g229800*, *Glyma.06 g146400*, *Glyma.06 g285800*, and *Glyma.20 g092200*, which encoded for IQ-domain, sulfate transporter, importin, 4-alpha-glucanotransferase, vascular plant one zinc finger protein, and 40S ribosomal protein (Table 3).

A total of 13 candidate genes associated with leaf chlorophyll content for the SCN-infected plants were identified (Table 3). Of the 13 reported candidate genes, 10 had functional annotations and 2 encoded for proteins with unknown functions. These candidate genes were involved in biomolecule transporters such as importin, transcription factors such as sequence-specific DNA binding transcription factors, and plant hormones-induced genes such as ethylene-responsive element binding factor. Candidate genes encoding for a leucine-rich repeat protein have been also identified. The 10-kb flanking regions of the most significant SNPs, Gm06_50,593,128_T_G, Gm15_43,797,502_G_T, Gm18_1,620,585_T_C, and Gm19_39,863,286_G_T, harbored genes such as *Glyma.06 g317100*, *Glyma.15 g233100*, *Glyma.18 g022100*, and *Glyma.19 g137300*, which encoded for biomolecule transporter, leucine-rich repeat-containing protein, BTB/POZ domain-containing protein, and Det1 complexing ubiquitin ligase, respectively (Table 3).

Results suggested a total of 15 candidate genes associated with chlorophyll content tolerance to SCN in soybean. Of the 15 candidate genes, 11 had functional annotations as reported in Table 3. Two genes, *Glyma.13 g294200* and *Glyma.15 g233100* encoding for a putative signaling peptide similar to TAX1 and a leucine-rich repeat-containing protein, respectively, were overlapping between CCI of SCN-infected plants and reduction in ICC. Most of the reported candidate genes encoded for molecule transporters within and between plant cells such as *Glyma.06 g317100*, *Glyma.07 g191600*, and *Glyma.13 g294200*. Candidate genes found within the most significant genomic regions containing the SNPs Gm13_39,378,998_G_A, Gm06_50,593,128_T_G, Gm07_35,908,169_T_C, Gm08_11,501,419_A_C, and Gm06_16,315,206_A_G were Glyma.13 g294200, Glyma.06 g317100, Glyma.07 g191600, Glyma.08 g149800, and Glyma.06 g187300, encoded for a putative signaling peptide similar to TAX1, protein transporter, secretory carrier membrane protein, Iron/ascorbate family oxidoreductases, and lipase (class 3) (Table 3).

### Marker-assisted selection accuracy and selection efficiency

SNP selection accuracy and efficiency pertaining to the significant SNPs were calculated for CCI under non-SCN infestation, CCI under SCN infestation, and reduction in CCI by SCN. For the plants under non-SCN infestation, selection accuracy varied from 35.94 to 87.80%, with an average of 55.40% (Table 4). The highest selection accuracy was found for the SNP Gm19_48,074,289_A_C (87.80%), whereas the SNP Gm20_1,621,036_T_C had the lowest selection accuracy (35.94%) (Table 4). Selection efficiency ranged from 25.56 to 54.55%, with an average of 35.71% (Table 4). The SNP Gm19_48,074,289_A_C (54.55%) had the highest selection efficiency. The lowest selection efficiency was found for the SNP Gm20_1,621,036_T_C (25.56%). Favorable alleles for the most significant SNPs Gm06_16,792,113_T_C, Gm20_1,621,036_T_C, Gm19_48,074,289_A_C, Gm06_11,948,808_G_A, and Gm06_47,439,414_C_T were T, T, C, G, and T, respectively (Table 4).

**Table 4:** Marker-assisted selection accuracy and efficiency for the significant SNPs associated with leaf chlorophyll content under non-SCN infestation

	Selection_accuracy_(%)^a^	Selection_efficiency_(%)^b^	Favorable_allele^c^
Gm04_2,574,201_T_G	44.32	30.23	G
Gm04_7,672,403_A_G	64.10	38.46	A
Gm05_40,299,923_A_G	50.00	33.77	G
Gm06_11,948,808_G_A	45.76	31.76	G
Gm06_16,792,113_T_C	43.88	28.10	T
Gm06_43,980,786_G_A	45.54	30.87	G
Gm06_47,439,414_C_T	49.02	32.05	T
Gm07_3,953,270_T_C	56.25	34.18	T
Gm07_3,990,308_A_G	56.00	34.15	A
Gm10_4,458,104_G_A	77.78	42.86	A
Gm10_41,610,215_C_T	50.65	33.91	T
Gm11_3,641,716_A_C	48.53	31.43	C
Gm11_4,702,578_C_A	41.18	27.18	A
Gm11_15,558,504_T_C	66.67	42.31	T
Gm11_37,978,746_G_T	56.98	37.40	G
Gm11_38,183,607_G_A	58.62	39.53	G
Gm12_1,460,019_T_C	49.00	34.75	T
Gm13_38,032,737_G_A	71.79	45.90	A
Gm19_42,195,616_G_A	62.90	39.80	G
Gm19_48,074,289_A_C	87.80	54.55	C
Gm20_1,621,036_T_C	35.94	25.56	T
Gm20_33,580,029_C_T	56.00	36.84	C

^a^Selection accuracy = 100*[(Number of genotypes having high leaf chlorophyll content with the favorable SNP allele)/(Number of genotypes having high leaf chlorophyll content with the favorable SNP allele + Number of genotypes having low leaf chlorophyll content with the favorable SNP allele)

^b^Selection efficiency = 100*[(Number of genotypes having high leaf chlorophyll content with the favorable SNP allele)/(Total number of genotypes having the favorable SNP allele)]

^c^Favorable allele corresponds to the allele with the highest frequency in the top 57 genotypes having the highest chlorophyll content under non-SCN infestation

Significant SNPs associated with CCI under SCN infestation exhibited a large variation in selection accuracy and selection efficiency. Selection accuracy was in the range of 41.18 to 85.11%, with an average of 56.01% (Table 5). Among the significant SNPs, the highest selection accuracy was recorded for Gm19_48,074,289_A_C (85.11%), whereas the lowest one was found for Gm18_1,620,585_T_C (41.18%) (Table 5). Selection efficiency varied from 26.72 to 60.61%, with an average of 38.24%. The SNP Gm19_48,074,289_A_C (60.61%) had the highest selection efficiency, whereas the SNP Gm18_1,620,585_T_C (26.72%) exhibited the lowest selection efficiency. Favorable alleles for the most significant SNPs, Gm06_50,593,128_T_G, Gm15_43,797,502_G_T, Gm18_1,620,585_T_C, Gm19_39,863,286_G_T, and Gm02_2,246,479_A_G associated with CCI under SCN infestation were T, T, T, G, and G respectively (Table 5).

**Table 5:** Marker-assisted selection accuracy and efficiency for the significant SNPs associated with leaf chlorophyll content under SCN infestation

	Selection_accuracy_(%)^a^	Selection_efficiency_(%)^b^	Favorable_allele^c^
Gm02_207,506_A_G	48.08	32.26	A
Gm02_2,246,479_A_G	66.67	42.50	G
Gm03_36,634,361_G_A	50.49	33.77	G
Gm05_39,995,603_C_T	51.02	33.56	C
Gm06_50,593,128_T_G	60.29	39.42	T
Gm07_11,956,773_T_C	50.00	34.02	C
Gm10_6,196,864_T_G	63.16	41.38	G
Gm13_39,378,998_G_A	54.37	35.44	G
Gm14_49,357,738_A_G	46.00	30.87	A
Gm15_43,797,502_G_T	68.18	45.45	T
Gm18_1,620,585_T_C	41.18	26.72	T
Gm19_38,917,571_A_G	53.85	34.43	A
Gm19_39,863,286_G_T	45.71	45.00	G
Gm19_48,074,289_A_C	85.11	60.61	C

^a^Selection accuracy = 100*[(Number of genotypes having high leaf chlorophyll content with the favorable SNP allele)/(Number of genotypes having high leaf chlorophyll content with the favorable SNP allele + Number of genotypes having low leaf chlorophyll content with the favorable SNP allele)

^b^Selection efficienty = 100*[(Number of genotypes having high leaf chlorophyll content with the favorable SNP allele)/(Total number of genotypes having the favorable SNP allele)]

^c^Favorable allele corresponds to the allele with the highest frequency in the top 57 genotypes having the highest chlorophyll content under SCN infestation

Overall, selection efficiency and accuracy of the SNPs associated with reduction in CCI were lower than those of the SNPs associated with CCI for the non-SCN-infected plants and SCN-infected plants. For the reduction in CCI, selection accuracy was in the range of 44.07 and 68.48%, with an average of 54.56% (Table 6). The SNP Gm15_43,797,502_G_T had the highest selection accuracy (68.18%), whereas the SNP Gm03_3,334,303_C_A showed the lowest selection accuracy (44.07%). SNP selection efficiency varied from 29.55 to 45.45%, with an average of 35.75% (Table 6). The SNP with the highest selection efficiency was Gm15_43,797,502_G_T (45.45%), whereas the one with the lowest selection efficiency was Gm03_3,334,303_C_A (29.55%). Favorable alleles corresponding to the most significant SNPs Gm13_39,378,998_G_A, Gm06_50,593,128_T_G, Gm07_35,908,169_T_C, Gm08_11,501,419_A_C, and Gm04_5,172,181_A_G were G, T, C, A, and A (Table 6).

**Table 6:** Marker-assisted selection accuracy and efficiency for the significant SNPs associated with decrease in leaf chlorophyll content under SCN infestation

	Selection_accuracy_(%)^a^	Selection_efficiency_(%)^b^	Favorable_allele^c^
Gm02_6,340,233_C_A	53.40	35.03	C
Gm03_3,334,303_C_A	44.07	29.55	A
Gm03_39,574,966_T_C	60.71	37.36	C
Gm04_5,172,181_A_G	61.54	41.67	A
Gm06_16,315,206_A_G	57.14	37.33	A
Gm06_50,593,128_T_G	60.29	39.42	T
Gm07_35,908,169_T_C	57.33	35.54	C
Gm08_9,848,168_T_C	50.96	33.13	T
Gm08_10,116,360_C_T	51.46	33.54	C
Gm08_11,501,419_A_C	50.00	33.54	A
Gm08_43,787,988_G_A	55.68	35.77	G
Gm09_6,664,095_T_C	47.06	31.17	C
Gm13_5,211,326_T_C	47.37	33.33	C
Gm13_39,378,998_G_A	54.37	35.44	G
Gm15_43,797,502_G_T	68.18	45.45	T
Gm18_1,427,298_G_T	53.33	34.78	G

^a^Selection accuracy = 100*[(Number of genotypes having the lowest decrease in leaf chlorophyll content with the favorable SNP allele)/(Number of genotypes having the lowest decrease in leaf chlorophyll content with the favorable SNP allele + Number of genotypes having the highest leaf chlorophyll content with the favorable SNP allele)

^b^Selection efficienty = 100*[(Number of genotypes having the lowest decrease in leaf chlorophyll content with the favorable SNP allele)/(Total number of genotypes having the favorable SNP allele)]

^c^Favorable allele corresponds to the allele with the highest frequency in the top 57 genotypes having the decrease in leaf chlorophyll content under SCN infestation

### Genomic selection (GS)

**Fig. 3:**
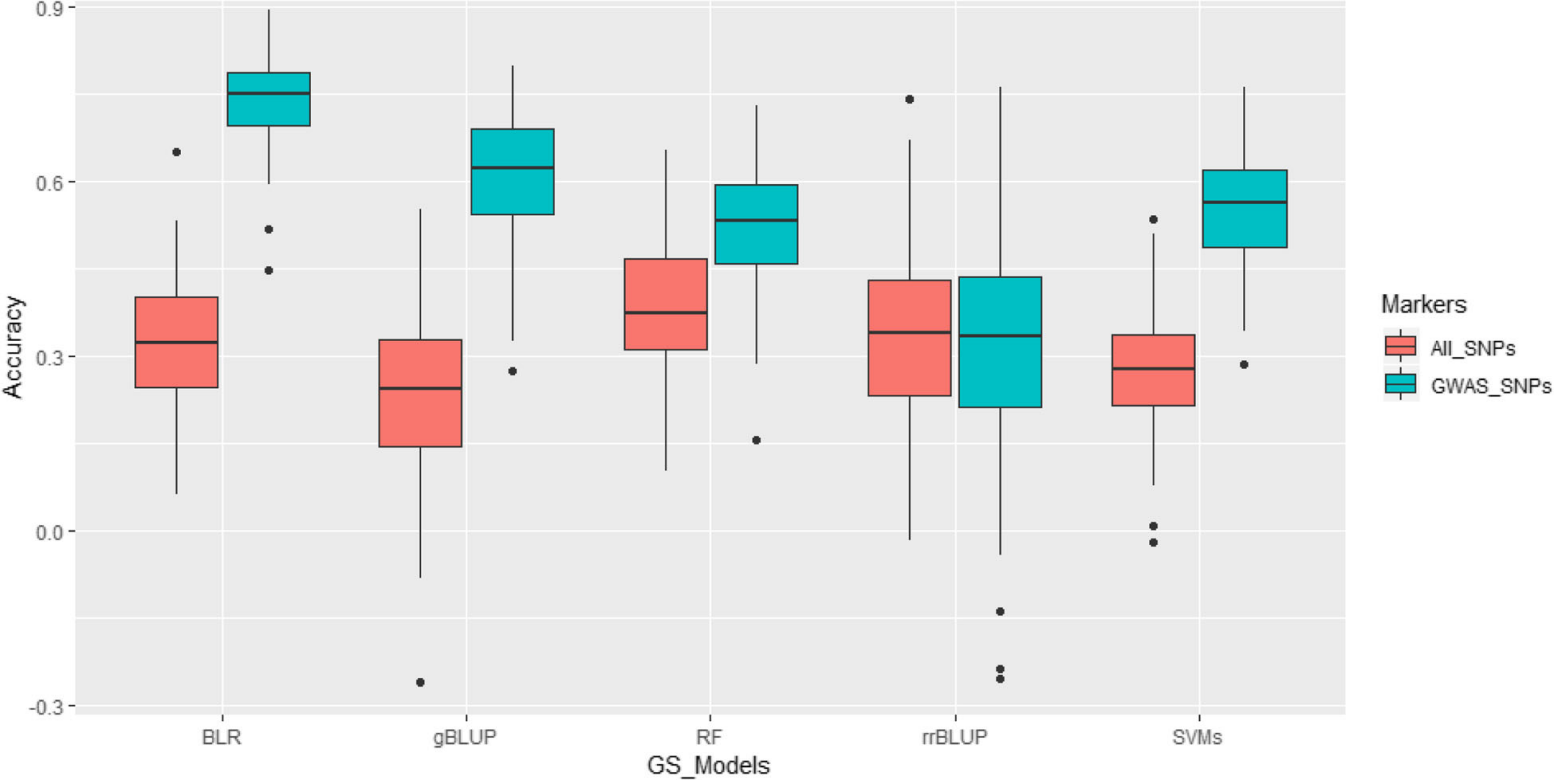
Genomic selection accuracy under 5 GS models for leaf chlorophyll content indices for plants without SCN infection

Genomic selection for CCI of non-SCN-infected plants, CCI of the SCN-infested plants, and reduction in CCI by SCN was conducted using 5 different statistical models. For the plants without SCN infection, average GS accuracy was 0.33, 0.23, 0.32, 0.38, and 0.28 for rrBLUP, gBLUP, Bayesian LASSO (BLR), Random Forest (RF), and Support Vector Machines (SVMs), respectively, when all 4089 SNPs were included in the models (Additional file 2: Table S2). Increase in GS accuracy was identified using GWAS-derived SNP markers for most of the statistical models expect rrBLUP. The highest increase was found when gBLUP was used (Fig. 3). When only significant SNPs were incorporated into the GS models, the Bayesian LASSO model provided the highest average GS accuracy (0.74), whereas the lowest one was recorded when rrBLUP was used (0.31), indicative of the GS accuracy being both SNP type and GS model-sensitive.

**Fig. 4:**
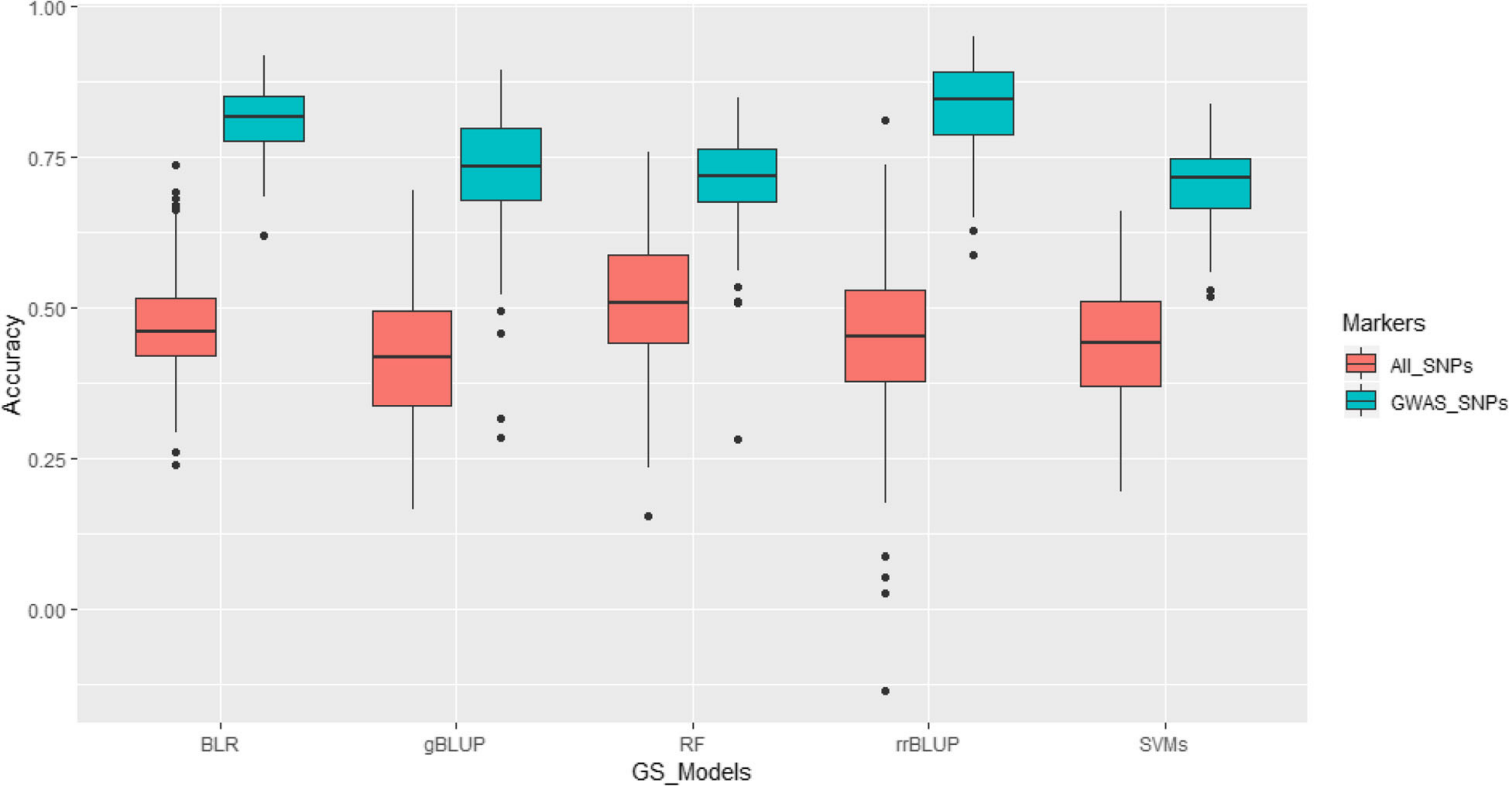
Genomic selection accuracy under 5 GS models for leaf chlorophyll content indices of the SCN-infected plants

For CCI under SCN infestation, GS accuracy was 0.45, 0.41, 0.47, 0.51, and 0.44 for rrBLUP, gBLUP, BLR, RF, and SVMs (Additional file 2: Table S2), respectively, when all SNPs were used to estimate the genomic estimated breeding values (GEBVs). In contrast to the results found for CCI under non-SCN infestation, GS increased by at least 39% when significant SNPs obtained from GWAS were used. Interestingly, the highest increase was found when rrBLUP was used (Fig. 4), and GS accuracy was the highest under the rrBLUP model (0.83), the second highest GS accuracy was provided by the Bayesian LASSO model (0.81), whereas SVMs showed the lowest GS accuracy (0.70). These results suggested that using significant SNPs obtained from GWAS could provide a better GS accuracy.

**Fig. 5:**
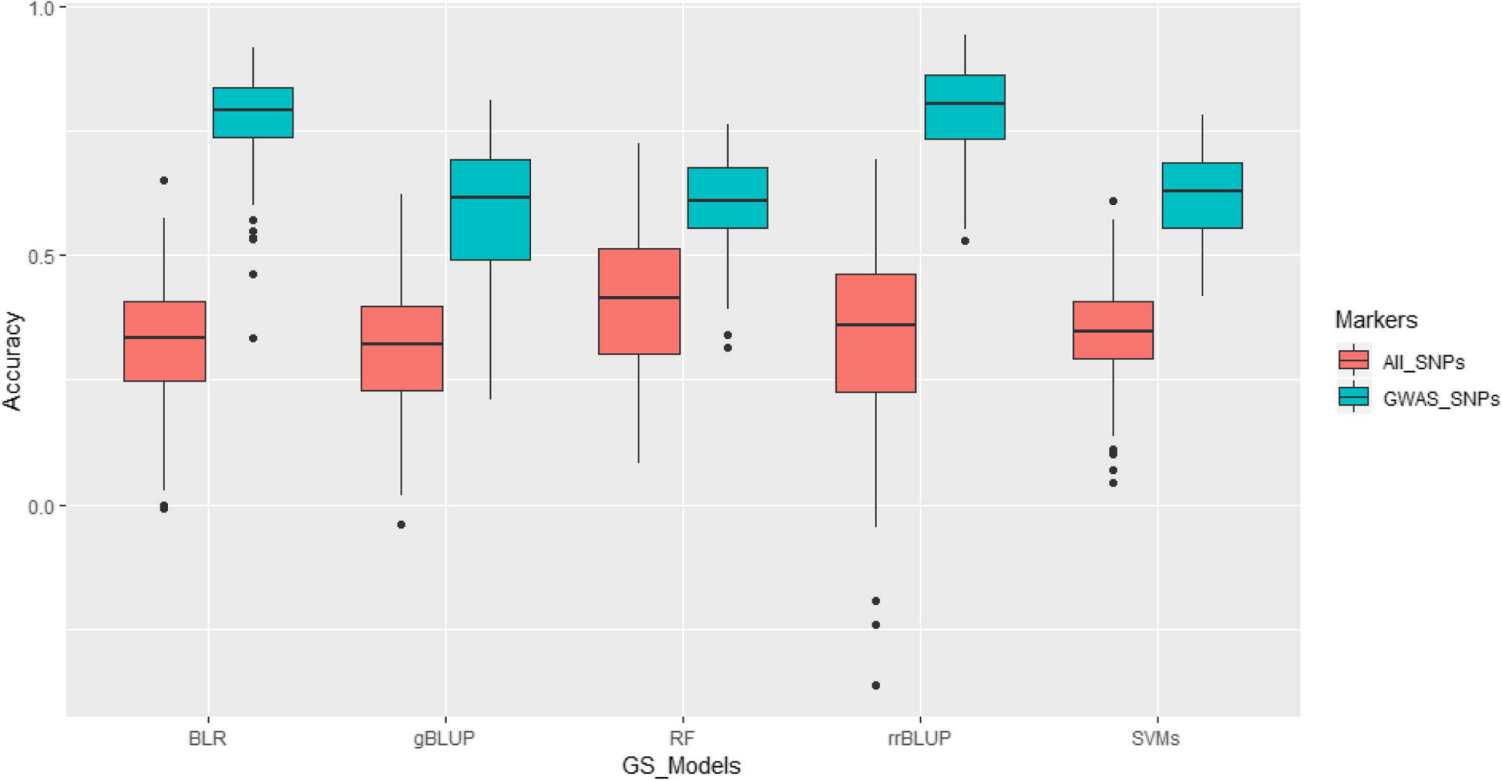
Genomic selection accuracy under 5 GS models for reduction in leaf chlorophyll content indices by SCN

GS accuracy for reduction in CCI by SCN was established. When all SNPs were used, the RF model exhibited the highest GS accuracy (0.41), whereas the lowest one was found under both rrBLUP and BLR (Additional file 2: Table S2). Significant increase in GS accuracy was found when only significant SNPs were considered (Fig. 5), which was similar to what was found for the two aforementioned traits. By only using GWAS-derived SNPs, GS accuracy was 0.79, 0.59, 0.77, 0.61, and 0.62 for rrBLUP, gBLUP, BLR, RF, and SVMs, respectively (Additional file 2: Table S2).

## Discussion

SCN resistance has been evaluated based on female (cyst) counts as measurements of SCN reproduction in soybean-infected plants. In this investigation, we evaluated tolerance of soybean to SCN based on leaf chlorophyll content. One pathway of SCN damage to soybean is reduction of chlorophyll content and induction of chlorotic symptoms [4]. However the molecular mechanisms involved in reduction of chlorophyll content and induction of chlorosis by SCN infection have not been studied. As far as we know, this investigation represents the first study of QTL associated with soybean chlorophyll content tolerance to SCN. Leaf chlorophyll content-based phenotyping strategy for SCN infection evaluation would allow for potential discovery of new loci associated with SCN tolerance, therefore making the genetic background broader for managing SCN, especially in the situation of the increasing SCN virulence. However, soybean tolerance to SCN should be based on yield response, and chlorophyll content can be one of factors contributing to soybean yield response [26]. Additional studies would definitely be required to establish a possible link between yield loss and reduction in chlorophyll under SCN infestation.

GWAS was performed in efforts to identify new loci conferring tolerance of soybean to SCN based on the assessment of reduction in leaf chlorophyll content, thus contributing to diversifying genes for SCN management. The use of GWAS to discover loci associated with SCN resistance has been shown to be promising in other studies [12, 22–24]. All previously reported GWAS investigations relied on mature female count to assess resistance to SCN and SNPs were associated with the female count. In this report, a total of 14 and 16 SNPs were found to be associated with CCI under SCN infestation and reduction in CCI by SCN infection, respectively. The significant SNP, Gm18_1,620,585_T_C, found on chromosome 18 and associated with CCI under SCN infestation was located at 88 Kbp upstream of the *rhg1* locus. In addition, the SNP Gm18_1,427,298_G_T mapped on chromosome 18 and significantly associated with a reduction of CCI was located at 281 Kbp upstream from the *rhg1* locus. These results indicate that this panel carries PI 88788 SCN-type resistance. Similar results were reported [12] stating that SNP markers located at even about 1 Mb from the *rhg1* locus were still in high LD with that SCN-resistant locus. In addition, a SNP marker located at 23 Kbp from Gm18_1,620,585_T_C and being tightly linked with the *rhg1* locus was reported [23]. Therefore, the two aforementioned SNPs which were found at a distance less than 300 Kbp from the *rhg 1* locus could be used in marker-assisted selection for SCN resistance. This finding suggested that assessing soybean tolerance to SCN based on chlorophyll reduction could provide useful result for selecting SCN-tolerant genotypes. Most of the significant SNPs associated with both CCI under SCN and reduction of CCI by SCN were found within previously reported SCN-resistant QTL and loci [12, 23, 24, 27–31]. In addition, the results suggested three new loci associated with chlorophyll content tolerance to SCN, of which, two were found on chromosome 3 and associated with the SNPs Gm03_3,334,303_C_A and Gm03_39,574,966_T_C, and the third one was mapped on chromosome 6 and associated with the SNP Gm06_50,593,128_T_G. The discovery of these new loci would permit for diversifying SCN-tolerance genes for SCN management.

Selection efficiency and accuracy were computed for the most significant SNPs associated with CCI under non-SCN infestation, CCI for the SCN-infected plants, and reduction in CCI as reported in Tables 4, 5, and 6. The use of SNP selection and accuracy has been highlighted in other GWAS-related reports [32, 33]. SNP selection accuracy and efficiency varied from medium to high in this study. This suggested that the significant SNPs identified from this investigation can be used for further marker-assisted selection for enhancing soybean resistance/tolerance to SCN.

Candidate genes found within a 10-kb region harboring significant SNPs have been established in Table 3. Candidate genes associated with CCI under non-SCN infestation encoded for proteins that were relevant to chlorophyll pathway. Functional annotation of the identified candidate genes consisted of chlorophyll A-B binding protein and 4-alpha-glucanotransferase found in photosynthetic leaves [34]. Proteins involved in plant development such as ROP interactive partner [35], formin-related [36], homeobox transcription factor [37], and uridine kinase [38] were identified as well. In addition, genes encoding for proteins associated with plant nutrition such as asparagine synthetase [39] and sulfate transporter [40] were found. The results were indicative of the robustness of the significant SNPs reported in this study since they permitted the discovery of candidate genes relevant to chlorophyll pathway and plant nutrition uptake under non-SCN infestation.

The genomic region harboring the overlapping SNP (Gm19_48,074,289_A_C), which was associated with both CCI under non-SCN infestation and SCN-infestation, enclosed a gene encoding for an importin, which was responsible for biomolecule trafficking within plant cells [41]. This gene could impact the flow of nutrients during plant development even under plant stress such as SCN infection. In addition, an overlapping candidate gene encoding for a leucine-rich repeat-containing protein (Table 3) was reported to enhance both leaf chlorophyll content under SCN infestation and tolerance to SCN in leaf chlorophyll content. Leucine-rich repeat protein has been widely shown to be involved in plant resistance mechanism to pathogen attack [36]. Further investigations are required to validate the function of this gene and its involvement in SCN tolerance in soybean. In addition, a signaling peptide was found to be associated with chlorophyll content tolerance to SCN. Signaling peptides have been reported to be involved in plant development [42], suggesting that this gene could enhance plant survival under stress of SCN infestation. Protein transporters and membrane proteins were widely found to be involved in chlorophyll content tolerance to SCN in this study. These proteins enhance the flow of biomolecules and nutrients within and between cells [41], thus permitting plant survival under SCN infection. Moreover, proteins associated with plant hormone signaling such as ethylene-responsive element binding factor 13, BTB/POZ domain-containing protein, and F-box family protein were identified. These signaling proteins have been demonstrated to be directly involved in plant defense against pathogens [43].

The new locus found on chromosome 3 harbors a gene encoding cytochrome P450, which has been shown to contribute to both plant development and defense under pathogen attack [44]. Further analysis is needed to confirm the involvement of this gene in resistance/tolerance to SCN. A lipase (class 3) was also found on chromosome 6. Lipases have been demonstrated to assist with plant defense mechanism against pathogen [45]. In addition, a methyltransferase-like protein gene was identified in a genomic region belonging to chromosome 2, which was in the vicinity of a significant SNP (Gm03_3,334,303_C_A) associated with chlorophyll content tolerance to SCN. This protein modulates gene expression [44]. Additional studies are required to understand the involvement of this methyltransferase gene in enhancing chlorophyll content tolerance to SCN in soybean plants.

Genomic selection has recently become more and more popular in modern and large-scale crop breeding programs. Previous studies highlighted that GS allowed for a more robust prediction of genotypic values compared to QTL [46]. In addition, GS has been proven to be more powerful compared to Marker-Assisted Selection (MAS) when dealing with traits controlled by genes having small effects [47]. However, little has been done with respect to investigating GS accuracy for SCN resistance/tolerance. In this study, average GS accuracy among different GS models was 0.31, 0.46, and 0.35 for CCI under non-SCN infestation, CCI in the SCN-infected plants, and reduction of CCI by SCN when all marker sets were used. When GWAS-derived SNPs was used, average GS accuracy was 0.55, 0.76, and 0.68 for the three aforementioned traits. A GS accuracy averaging 0.46 for SCN resistance based on SCN female count and using different GS models was previously reported [12]. The results from the GS involving CCI was in agreement with that of found by previous investigations [12] despite the fact that two different phenotypes, leaf chlorophyll content and female count, were used. In addition, GS accuracy involving linear models (rrBLUP, gBLUP, RF) performed almost similarly as those using more sophisticated approach (Bayesian LASSO) and machine learning strategy (Additional file 2: Table S2). Similar findings were reported [12].

## Conclusions

A total of 172 soybean genotypes were evaluated for the effect of SCN HG Type 1.2.3.5.6.7 (race 4) on soybean leaf chlorophyll content in the greenhouse. The leaf chlorophyll content indices (CCI) were used as the phenotypic data and 4089 filtered and high-quality SNPs obtained from the Soy6K SNP Infinium Chips as the genotypic data for the GWAS and GS analysis. A total of 22, 14, and 16 SNP markers were associated with CCI of non-SCN-infected plants, SCN-infected plants, and reduction of CCI by SCN, respectively. The average GS accuracy ranged from 0.31 to 0.46 with all SNPs and varied from 0.55 to 0.76 when GWAS-derived SNP markers were used across five GS models. The SNP markers from this study could be used to improve the soybean leaf chlorophyll content tolerance to SCN infection through MAS and GS. Further study is needed to investigate the translation of a reduction in chlorophyll content to yield loss under SCN infestation.

## Methods

### Plant materials and phenotyping

A total of 172 soybean genotypes were used for this study (Additional file 1: Table S1). This panel of lines was part of the panel of 282 lines selected by Bao from the University of Minnesota soybean breeding program using PediTree for the previous study of association mapping (AM) and genomic selection (GS) of SCN resistance by Bao et al. (2015) [12]. Most of the lines were susceptible to SCN in terms of SCN female counts of HG Type 0 (race 3), but there were six resistant, six moderately resistant, and four moderately susceptible lines (Additional file 1: Table S1). The few resistant lines contained SCN resistance genes from PI 88788 [12]. The plant materials listed in the Additional file 1: Table S1 are preserved in the soybean breeding program in the University of Minnesota, and the lines with PI numbers are available in the United States Department of Agriculture Germplasm Resources Information Network (USDA GRIN).

The phenotyping experiment was conducted in the greenhouse at the University of Minnesota St. Paul campus. A soil without SCN infestation was collected from a soybean field. The soil was mixed and divided to four lots. For each replicate, a lot of soil was further thoroughly mixed, and then divided to 1-kg lots that were placed in 1-galon plastic bags. In each bag, 500 g sand were added for increase of drainage. The soil of each bag was used for one pot.

Soybean cyst nematode HG Type 1.2.3.5.6.7 (race 4) was cultured on susceptible soybean ‘Sturdy’. Race 4 can reproduce well on the lines containing resistance genes from PI 88788. Eggs were collected from the soybean roots and soil. The eggs (80,000 eggs/bag ≈ 10,000 eggs/100 cm^3^ soil) in 10 ml water were added into the soil in the plastic bag before planting. For the pots without SCN eggs, 10 ml water was added into the bags. The soil-sand with or without SCN eggs were mixed in each bag. About 83% of the soil from each bag was placed in a pot. Ten soybean seeds were placed on the surface each pot and the seeds were covered with the remaining soil. The pots were placed in the greenhouse benches. The two pots (SCN and no-SCN) of the same soybean line were placed together to minimize the environmental difference between the SCN and no-SCN treatments within a genotype. Each line was replicated four times. Due to the large number of lines, this experiment was conducted at four different times with approximately 60 lines per time in the same greenhouse. Although pots of each replicate were placed in a block, the experiment was considered randomized design because the lines were evaluated in four groups at four different times. Leaf chlorophyll content indices (CCI) were recorded using SPAD 502 DL Meter (Minolta) on the second trifoliate leaves of 8-week old soybean plants. A total of 15 observations were taken from the 15 leaflets of the five plants each pot, and the average CCI for each pot was calculated from the 15 observations.

Data analysis.

Data consisted of CCI without SCN, CCI of plants in soil infested with SCN, and percentage reduction in CCI. Percentage reduction in CCI was obtained as following:

- Leaf chlorophyll content Reduction = 100 x[(CCI without SCN - CCI of plants infected with SCN)/ CCI without SCN]

Descriptive statistics were generated using the option ‘Tabulate’ of JMP Genomics®7 (SAS Institute Inc., Cary, NC, USA). Data were visualized through combined violin and boxplots using the packages ‘ggplot2’, ‘labeling’ and ‘gridExtra’ of R 3.3.0. Data were analyzed using PROC GLM of SAS®. 9.4. The statistical model for the analysis was the following.

$$ {Y}_{ij}=\mu + Gi+{\varepsilon}_{ij}\; with\;i=1,2,...,172\; and\;j=1,2,3,4 $$

Y_ij_ denoted the response of the i^th^ genotype at the j^th^ replication, G_i_ represented the effect of the i^th^ genotype (assumed to have fixed effect), and ε_ij_ was the experimental error associated with the ij^th^ observation.

### Genotyping

DNA was extracted from young leaves of each accession using DNeasy 96 Plant Kit (QIAGEN, Valencia, CA). The DNA samples were genotyped using an Illumina GoldenGate SNP assay. A total of 4252 SNPs obtained from the Soy6K SNP Infinium Chips (https://www.soybase.org/snps/download.php) were used in the genotyping. SNP data having more than 10% missing data, more than 20% heterozygous SNPs, and minor allele frequency less than 4% were removed from the analysis. After SNP filtering, a total of 4089 high-quality SNPs were used for further analysis.

### Genome-wide association study (GWAS)

GWAS was performed using a Fixed and Random Model Circulating Probability Unification (FarmCPU) in R software as previously described [48]. FarmCPU was shown to have an enhanced statistical power when running for GWAS [49]. Both fixed (FEM) and random effects (REM) were included in the model and run iteratively until no new pseudo QTNs were established. The model was described as following [48].

- $$ \mathrm{FEM}:{\mathrm{y}}_{\mathrm{i}}={\mathrm{M}}_{\mathrm{i}1}{\mathrm{b}}_1+{\mathrm{M}}_{\mathrm{i}2}{\mathrm{b}}_2+...+{\mathrm{M}}_{\mathrm{i}\mathrm{j}}{\mathrm{b}}_{\mathrm{j}}+{\mathrm{S}}_{\mathrm{i}\mathrm{k}}{\mathrm{d}}_{\mathrm{k}}+{\mathrm{e}}_{\mathrm{i}} $$

where y_i_ represented the phenotypic data obtained from the i^th^ individual, M_ij_’s denoted the pseudo QTNs, b_j_’s were the effect of the j^th^ pseudo QTN, S_ik_ denoted the k^th^ SNP corresponding to the i^th^ individual, and e_i_ was the random error for the i^th^ observation such that e_i_ ~ *N*(0, σ^2^_e_).

(b) $$ \mathrm{REM}:{\mathrm{y}}_{\mathrm{i}}={\mathrm{u}}_{\mathrm{i}}+{\mathrm{e}}_{\mathrm{i}} $$

where y_i_ was the phenotype corresponding to the i^th^ individual, u_i_ denoted the total genetic effect (random effect) for the i^th^ individual with a variance-covariance matrix defined as 2Kσ^2^_a_, σ^2^_a_ was an unkown genetic variance and K was the Kinship generated from the pseudo QTNs, and e_i_ was the residual such that e_i_ ~ *N*(0, σ^2^_e_). Estimate of the variance-covariance matrix was computed using a Singular Value Decomposition (SVD) of the pseudo QTNs based upon the FaST-LMM (Factored Spectrally Transformed Linear Mixed Model) algorithm.

### Candidate gene(s) discovery

Significant SNPs (LOD > 2.0) [50] postulated from GWAS were used for candidate gene search. A 10-kb genomic region flanking each SNP was considered. Functional annotation of candidate genes was investigated in Soybase (www.soybase.org).

### SNP selection accuracy and selection efficiency

SNP selection accuracy and selection efficiency were computed based on the formulas previously developed [33]. The top 57 performers and the 57 least performers for each trait were chosen.

$$ Selection\ accuracy=100\ \mathrm{x}\ \Big[\left(\mathrm{Number}\ \mathrm{of}\ \mathrm{genotypes}\ \mathrm{having}\ \mathrm{high}\ \mathrm{CCI}\ \mathrm{with}\ \mathrm{the}\ \mathrm{favorable}\ \mathrm{SNP}\ \mathrm{allele}\right)/\left(\mathrm{Number}\ \mathrm{of}\ \mathrm{genotypes}\ \mathrm{having}\ \mathrm{high}\ \mathrm{CCI}\ \mathrm{with}\ \mathrm{the}\ \mathrm{favorable}\ \mathrm{SNP}\ \mathrm{allele}+\mathrm{Number}\ \mathrm{of}\ \mathrm{genotypes}\ \mathrm{having}\ \mathrm{low}\ \mathrm{CCI}\ \mathrm{with}\ \mathrm{the}\ \mathrm{favorable}\ \mathrm{SNP}\ \mathrm{allele}\right). $$

$$ Selection\ efficiency=100\ \mathrm{x}\ \left[\left(\mathrm{Number}\ \mathrm{of}\ \mathrm{genotypes}\ \mathrm{having}\ \mathrm{high}\ \mathrm{CCI}\ \mathrm{with}\ \mathrm{the}\ \mathrm{favorable}\ \mathrm{SNP}\ \mathrm{allele}\right)/\left(\mathrm{Total}\ \mathrm{number}\ \mathrm{of}\ \mathrm{genotypes}\ \mathrm{having}\ \mathrm{the}\ \mathrm{favorable}\ \mathrm{SNP}\ \mathrm{allele}\right)\right]. $$

### Genomic selection (GS)

Genomic selection was conducted using all 4089 SNPs and the SNPs showing association (LOD > 2.0) [50] with the traits of interest, respectively. Genomic estimated breeding value (GEBV) was estimated using 5 different statistical models described as following.

#### Ridge regression best linear unbiased predictor (rrBLUP)

The rrBLUP model was y = WGβ + ε [25] where y is the vector phenotype, β was the marker effect with β~*N*(0, Iσ^2^_β_), W was the incidence matrix relating the genotype to the phenotype, G was the genetic matrix, and ε was the random error. The solution for the rrBLUP equation was defined by β^=(Z^T^Z + Iλ)^−1^Z^T^y with Z = WG. The ridge parameter was described as λ = σ^2^_e_/σ^2^_β_ with σ^2^_e_ being the residual variance and σ^2^_β_ the marker effect variance. rrBLUP was performed using the ‘rrBLUP’ package of R [51].

#### Genomic best linear unbiased predictor (gBLUP) [52]

The gBLUP model was y_r_ = X_r_β + Z_r_μ_r_ + ε_r_ where the ‘r’ subscript referred to the genotypes involved in the reference panel, y_r_ was the vector phenotype, β was the genetic effect being assumed to be fixed, X_r_ was the incidence matrix relating β to y_r_, μ_r_ denoted the polygene random additive effect with μ_r_ ~*N*(0, Kσ^2^_a_) where K was the Kinship matrix and σ^2^_a_ the additive genetic variance, ε_r_ was the random error with ε_r_ ~*N*(0, *I*σ^2^_e_) where *I* was an identity matrix and σ^2^_e_ was the residual variance.

The Kinship matrix was divided into reference and inference panel as described below.

$$ K=\left(\begin{array}{ccc}{K}_{rr}& & {K}_{ri}\\ {}& & \\ {}{K}_{ir}& & {K}_{ii}\end{array}\right) $$

where K_rr_ was the variance-covariance matrix for the reference group, K_ii_ represented the variance-covariance matrix for the inference group, and K_ir_ = (K_ri_)’ denoted the covariance matrix between individuals from the reference and inference groups, respectively.

The predicted genetic effect in the inference panel was obtained using the following formula [53].

$$ {\mu}_i={K}_{ir}{\left({K}_{rr}\right)}^{-1}{\mu}_r $$

where u_i_ denoted the polygene effect in the inference group, and K_ir_, K_rr_, and μ_r_ were previously described. gBLUP was performed using GAPIT [54].

#### Bayesian least absolute shrinkage and selection operator (Bayesian LASSO)

Bayesian LASSO was a modified version of LASSO regression. In Bayesian LASSO, posteriors related to the genetic and residual variances were Exponential and Multivariate Normal, respectively. The statistical model was described as following [55].

y = μ + Xg + ε where y was the vector phenotype, μ denoted the overall mean, X represented the SNP matrix, g was the vector of random effect due to SNPs, ε represented the vector of random residuals, the posterior distribution of g was defined by g|λ~∏_j_(λ/2)exp.{− λ|g_j_|} with λ~Unif(0,1,000,000) being the λ prior, and the posterior distribution of ε|σ^2^_e_~*MVN*(0, *I*σ^2^_e_) with σ^2^_e_~Inv-χ^2^(4) being the prior distribution for σ^2^_e_. Bayesian LASSO was done in R using the package ‘BGLR’ [56] with burn-ins and iterations of Markov-Chain Monte Carlo (MCMC) equal to 5000 and 1000, respectively [57].

#### Random Forest

Random forest regression was based upon on unpruned tree decision [58]. In random forest regression, a new split was obtained from a Bootstrap sample generated from the training set. Splitting at the tree node level was based upon randomly selected subsets of predictors. The prediction of a new observation x((*F*^_rf_^B^(x)) was the mean outcomes obtained from B trees defined by {T(x, ψ_b_)}_1_^B^. Therefore, the prediction function was described as following.

*F*^=(1/B)* Σ_b_ T(x, ψ_b_) with b = 1….B and ψ_b_ denoted the b^th^ Random Forest tree defined by the split variables, cutpoints at each node, and the terminal node. Random Forest regression was established in R using the package ‘randomForest’ [59]. A total of 500 trees and 4 branches were used [12].

#### Support vector machines

Support Vector Machines (SVMs) have been recently widely used in genomic selection-related studies. This is a kernel-based supervised machine learning approach with a regression equation described as following [60].

y = f(X|β) + ε with f(X|β) = Σ_j_ β_j_ K_h_ (X, X_j_) being the kernel generating function. In this study, a Gaussian kernel was used. SVMs regression was performed in R using the package ‘kernlab’ [61].

### Cross-validation

A five-fold cross validation was performed for the genomic selection study [62]. The association panel was randomly divided into 5 disjoint groups. A total of 4 subsets were used as training set, and the remaining set was considered testing set. A total of 100 replications were conducted at each fold. Mean and standard errors corresponding to each fold were computed. Genomic selection accuracy was obtained by computing the Pearson’s correlation coefficient between GEBV and the observed phenotype for the testing set as previously described by [62].

## Supplementary information

**Additional file 1: Table S1.** Descriptive statistics for leaf chlorophyll content indices (CCI) of plants grown in soils without the soybean cyst nematode (SCN), CCI of plants in soils infested with SCN, and reduction in CCI by SCN.

**Additional file 2: Table S2.** Genomic selection accuracy for leaf chlorophyll content indices (CCI) without the soybean cyst nematode (SCN) infestation, CCI of the SCN-infested plants, and reduction in CCI by SCN.

## Data Availability

All raw data for this article are provided in the Additional files 1 and 2.
